# Combination of Multidisciplinary Therapies Successfully Treated Refractory Ventricular Arrhythmia in a STEMI Patient: Case Report and Literature Review

**DOI:** 10.3390/healthcare10030507

**Published:** 2022-03-10

**Authors:** Nung-Sheng Lin, Yen-Yue Lin, Yung-Hsi Kao, Chih-Pin Chuu, Kuo-An Wu, Jenq-Shyong Chan, Po-Jen Hsiao

**Affiliations:** 1 Department of Emergency, Taoyuan Armed Forces General Hospital, Taoyuan 325, Taiwan; wildlouis02@gmail.com; 2 Department of Emergency, Tri-Service General Hospital, National Defense Medical Center, Taipei 114, Taiwan; 3 Department of Life Sciences, National Central University, Taoyuan 320, Taiwan; ykao@cc.ncu.edu.tw; 4 Institute of Cellular and System Medicine, National Health Research Institutes, Miaoli 350, Taiwan; cpchuu@nhri.org.tw; 5 Graduate Program for Aging, China Medical University, Taichung 404, Taiwan; 6 Division of Pulmonary & Critical Care Medicine, Department of Internal Medicine, Taoyuan Armed Forces General Hospital, Taoyuan 325, Taiwan; wu5830@ms26.hinet.net; 7 Division of Nephrology, Department of Internal Medicine, Taoyuan Armed Forces General Hospital, Taoyuan 325, Taiwan; jschan0908@yahoo.com.tw; 8 Division of Nephrology, Department of Internal Medicine, Tri-Service General Hospital, National Defense Medical Center, Taipei 114, Taiwan; 9 Division of Nephrology, Department of Medicine, Fu-Jen Catholic University Hospital, School of Medicine, Fu-Jen Catholic University, New Taipei City 242, Taiwan

**Keywords:** double sequential defibrillation, refractory ventricular fibrillation, pulseless ventricular tachycardia, out-of-hospital cardiac arrest

## Abstract

Ventricular fibrillation (VF) is a life-threatening cardiac arrhythmia that can lead to loss of cardiac function and sudden cardiac death. The most common cause of VF is ischemic cardiomyopathy, especially in the context of an acute coronary event. Prompt treatment with resuscitation and defibrillation can be lifesaving. Refractory VF, or pulseless ventricular tachycardia (pVT), refers to cases that do not respond to traditional advanced cardiac life-support (ACLS) measures, and it has a low survival rate. Some new life-saving interventions and novel techniques have been proposed as viable treatment options for patients presenting with refractory VF/pVT out-of-hospital cardiac arrest; these include extracorporeal membrane oxygenation (ECMO), esmolol, stellate ganglion block (SGB), and double sequential defibrillation (DSD). Recently, DSD has been discussed and used more frequently, but its survival rate is still not promising. We report a case of refractory VF caused by acute myocardial infarction that was treated with ACLS, DSD, ECMO, and cardiac catheterization in sequence, with a successful outcome.

## 1. Introduction

Normal cardiac electrophysiology requires intricate, spontaneous, automatic, and synchronized signalling within cardiomyocytes. When ventricular cardiomyocytes endure damage, they can become prone to electrical hyperexcitability and depolarization without a signal from the sinoatrial node, leading to ventricular fibrillation (VF) [1]. Current management guidelines for VF put great emphasis on the importance of early, high-quality cardiopulmonary resuscitation (CPR), ventilatory support, defibrillation, and drug therapies [2]. Refractory VF, or pulseless ventricular tachycardia (pVT), refers to cases that do not respond to traditional advanced resuscitation, and they have a significantly low survival rate [3]. If their circulation is not recovered immediately, VF patients will have an ominous outcome. Although many interventions and novel techniques have been proposed as viable treatment options for patients presenting with refractory arrhythmia, no treatment has a promisingly high efficacy rate [4]. We present a patient with refractory VF who was successfully treated with a combination of modalities. This report also provides an update and review on the management of refractory VF/pVT, including electrical management (DSD), mechanical support, drugs, etc.

## 2. Case Presentation

**Figure 1:**
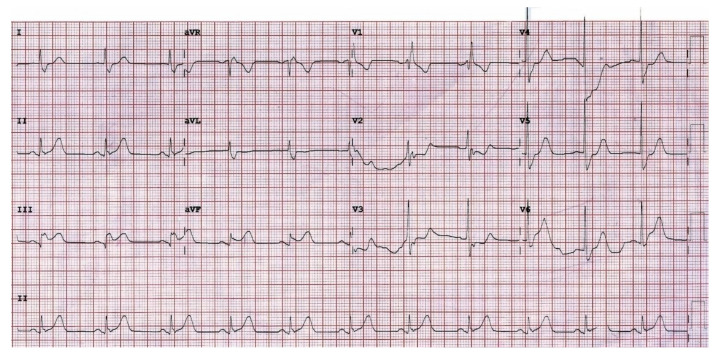
Twelve-lead electrocardiograms on admission. Right bundle branch block and elevation of the ST segment in leads III, and aVF.

**Figure 2:**
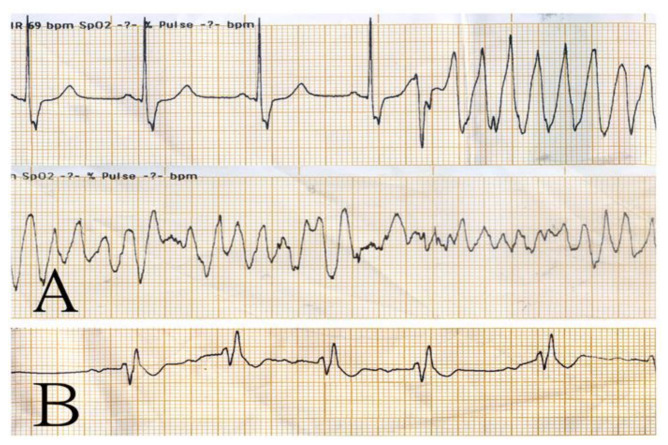
ECG strips were obtained when the patient collapsed (**A**). After two rounds of double sequential defibrillation (**B**), the ECG started to show ventricular defibrillation and returned to sinus rhythm.

A 65-year-old man presented to the emergency department (ED) because of chest pain and marked diaphoresis for one hour. He had a history of coronary artery disease after percutaneous coronary intervention approximately one year prior. On arrival, he was agitated and hypotensive (98/60 mmHg). Oxygen supplements, aspirin, and fluid challenge were given to the patient immediately. A 12-lead electrocardiogram (ECG) revealed ST-segment elevation in leads III, and aVF (Figure 1) suggesting acute inferior myocardial infarction, and a right-side EKG revealed no ST elevation in lead V4. Auscultation of the chest and heart showed clear breathing sounds and an absence of cardiac murmurs, and there was no sign of jugular vein distension besides wet skin. The laboratory findings revealed that cardiac enzymes, serum electrolytes, and liver function tests were within normal limits, except for slight abnormal renal function (Table 1). The cardiology specialist of our hospital was consulted for emergent percutaneous coronary intervention. Anticoagulation with heparin was given at that time. However, the patient’s consciousness changed abruptly and sustained collapse, and monitoring of the defibrillator showed VF (Figure 2A). Mechanical cardiopulmonary resuscitation (CPR) was performed continually, and defibrillation was performed with biphasic shocks of 200 joules (J). Endotracheal intubation was performed, and intravenous epinephrine and amiodarone were administered. The patient’s heart rhythm still showed VF after five cardiac defibrillations. Double sequential defibrillation (DSD) was performed twice, with two sets of pads placed in the anterolateral and anteroposterior positions. The patient had recovered sinus rhythm (Figure 2B) and returned to spontaneous circulation (ROSC). Extracorporeal membrane oxygenation (ECMO) was performed on the right femoral artery. The patient was then sent to the cardiac catheterization laboratory, which revealed that the left main coronary artery was patent; the previously implanted proximal right coronary artery and middle left anterior descending artery stent had 30% and 90% restenosis, respectively; and there was 90% occlusion over the middle left circumflex artery. Percutaneous coronary intervention was performed with two stents, respectively placed at the middle left anterior descending artery and middle left circumflex artery. The patient was transferred to the intensive care unit, and ECMO was stopped on the third day of admission. His highest level of high-sensitivity troponin-I level was 31478.7 mg/dL (normal <10 mg/dL), and declined gradually during hospitalization. He was extubated three days later and discharged after 13 days in hospital. His cardiac echography showed an estimated ejection fraction of 73% using the Teichholz method. At a follow-up clinic visit 3 months later, the patient was still doing well.

## 3. Discussion

### 3.1. Definition and Incidence of Refractory VF/pVT

Our patient presented at our ED with ST-elevation and myocardial infarction (MI), and developed refractory VF. Out-of-hospital cardiac arrest (OHCA) is the loss of mechanical cardiac function and the absence of systemic circulation outside of the hospital setting. Among patients with OHCA, over 20% present with a shockable rhythm, such as VF/pVT [3,5]. VF/pVT is associated with higher survival and predicts neurologically intact survival compared with non-shockable cardiac arrest rhythms (pulseless electrical activity and asystole) [6]. A subset of these patients are still not successfully resuscitated by standard ACLS care and remain in VF/pVT, with an incidence ranging from 2% to 28% [3]. Normal cardiac electrophysiology requires intricate, spontaneous, automatic, and synchronized signalling within cardiomyocytes. When ventricular cardiomyocytes endure damage, they can become prone to electrical hyperexcitability and depolarization without a signal from the sinoatrial node, leading to VF. The usual cause of ventricular cardiomyocyte injury is an ischaemic event such as MI. Without intervention, the VF rhythm eventually progresses to asystole and death [1]. Refractory VF/pVT is defined as persistent dysrhythmia despite ACLS and at least three consecutive standard defibrillation (SD) attempts [4]. The annual incidence of refractory VF can be up to 0.6 cases per 100,000 people, and its mortality can be as high as 97% [6].

### 3.2. Treatment Categories of Refractory VF/pVT

The mainstays of treatment for refractory VF/pVT remain ACLS and early SD [7]. Some new life-saving interventions and novel techniques have been proposed as viable treatment options for patients presenting with refractory VF/pVT OHCA, including epinephrine, amiodarone, lidocaine, magnesium sulfate, beta-blocker, nitrate, stellate ganglionic block, ECMO, and DSD [4]. They can be categorized into four types (Table 2).

#### 3.2.1. Electrical Defibrillation

**Figure 3:**
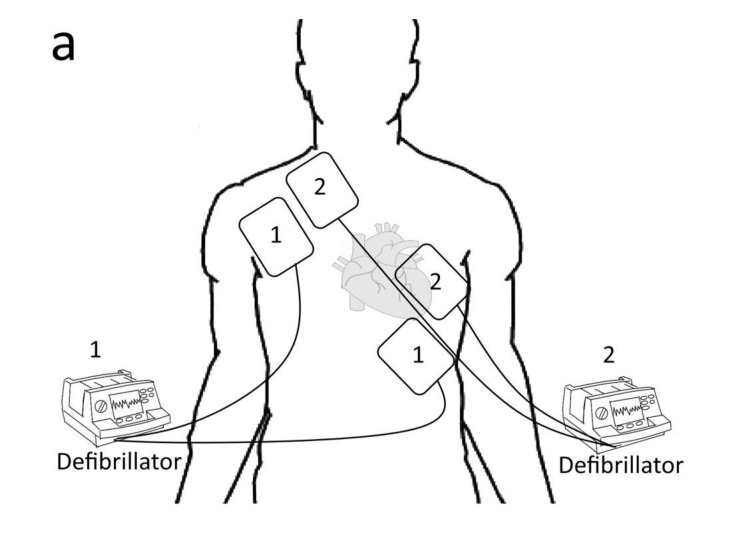

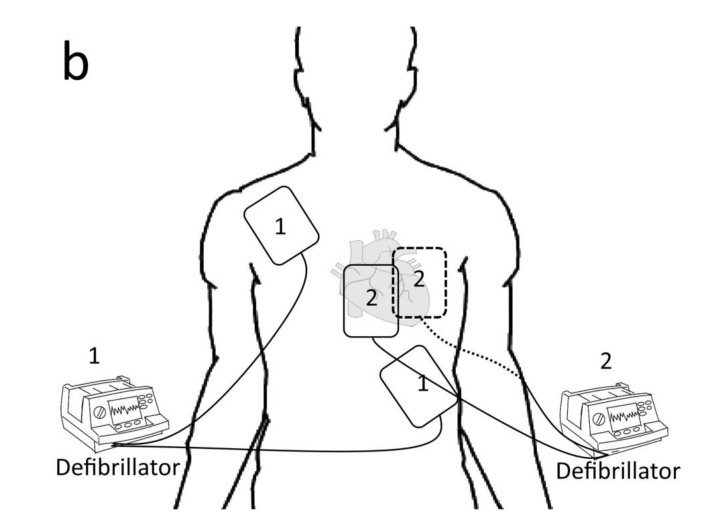
Two types of setting in DSD: (**a**) place another set of pads next to the original set in an anterior–lateral position; (**b**) place another set of pads in anterior–posterior position, with the anterior pad at the precordium region, and the posterior pad at the left or right infrascapular region.

The first category is electrical defibrillation, which includes SD and DSD. SD is a standard treatment for life-threatening cardiac dysrhythmias, specifically VF and pVT. The exact mechanism by which defibrillation produces effects is not well understood. One theory is that successful defibrillation affects most of the heart, resulting in too-little heart muscle still being malfunctional for the arrhythmia to continue. Recent mathematical models of defibrillation have provided new insight into how cardiac tissue responds to strong electrical shock [11]. DSD was recently the use of two defibrillators simultaneously at their highest allowed energy setting to treat refractory VF. There are currently two types of setting for pad placement (Figure 3): the first type involves placing one set of pads in the anterior–posterior position, and the second set of pads (second defibrillator) in the anterior–lateral position; the second type involves placing another set of pads next to the original set in an anterior–lateral position. Both defibrillation buttons are pressed simultaneously [12]. There are a few theories as to why DSD is effective. First, higher energy is delivered to overcome the increasing defibrillatory threshold. Next, the first shock lowers the defibrillation threshold, thus increasing the second shock’s success. It is also suggested that alternate vectors of defibrillation provided by the second set of pads may be more likely to stop the myocytes fibrillating [4]. Cheskes et al. [13] published a study on Resuscitation that revealed that DSD may be time-sensitive. Early defibrillation attempts (SD is attempted 4-8 times) are considered to have higher VF-termination and ROSC rates [12]. In our patient, we performed early DSD after three SD attempts, which could be one reason for his ROSC. The only randomized controlled trial (RCT) was conducted by Cheskes et al. in 2020. It was an internal pilot study. The study only reported short-term outcomes (ROSC and VF termination), and it showed different effect estimates compared to most other studies. The full RCT will be completed in September, 2022 (clinicaltrials.gov: NCT04080986) [13]. A systematic review was published by Charles D. Deakin et al. on resuscitation in 2020. It showed that DSD was not associated with improved outcomes from OHCA. However, there were some limitations to their interpretation. Further high-quality evidence will be needed to answer some important questions, such as the optimal pad placement, the timing of DSD, the interval between shocks, and the optimal energy settings [14].

#### 3.2.2. Mechanical Support

The second category is mechanical support for circulation, including high-quality CPR and ECMO. CPR alone is unlikely to restart the heart. Its main purpose is to restore the partial flow of oxygenated blood to the brain and heart. Immediate CPR followed by defibrillation within 3–5 min of sudden VF cardiac arrest dramatically improves survival. In 2015, the Minnesota Resuscitation Consortium (MRC) implemented an advanced perfusion and reperfusion life-support protocol to improve outcomes of refractory VF/pVT OHCA [15]. The ARREST trial, published in the Lancet in 2020, revealed significant survival benefits [10]. Although our patient experienced cardiac arrest in hospital, high-quality mechanical CPR was performed. The patient also met all the inclusion criteria and did not meet any of the exclusion criteria. The MRC VF/pVT protocol may also have been suitable for this patient. Additionally, ECMO protected the patient’s circulation from the possibility of recurrent VF/pVT during transportation from the ED to the cardiac catheterization laboratory (CCL).

#### 3.2.3. Medical Treatments

The third category is medical treatment, including epinephrine, amiodarone, lidocaine, magnesium sulfate, beta-blockers, and nitrate. Epinephrine was administered after at least one attempt at defibrillation and 2 min of CPR. Premature treatment with epinephrine (within two minutes of defibrillation) has been associated with decreased survival. According to the American Heart Association update, amiodarone and lidocaine are considered to be particularly useful for patients with witnessed arrest. Lidocaine brings a higher rate of survival to hospital admission and a high ROSC rate. Magnesium sulfate is not recommended for routine use except for torsades de pointes [2]. Beta-blockers were not included as a drug of choice in the previous recommendation, but a recent systematic review and meta-analysis (2020) showed that beta-blockers may be associated with improved outcomes, including ROSC, survival to discharge, survival to admission, and overall survival, with a favourable neurologic outcome. Still, no RCTs have identified any beta-blockers for refractory OHCA [3]. Other anti-arrhythmic agents, such as Sotalol and Mexiletine, are not universally available and have no reported use for refractory VF/pVT. On the other hand, deep sedation was mentioned as potentially being helpful in the AHA 2017 Guidelines and ESC 2015 Guidelines for the management of patients with ventricular arrhythmias. The recommendations in the guidelines are unreferenced. A small study by Bundgarrd et al. suggested that deep sedation and intubation reduced or terminated VT in patients with refractory to guideline-recommended usual clinical care in the acute setting [9]. It can be used as a bridge to definitive treatment in patients with different underlying cardiac disorders. Bundgarrd et al. preferred using propofol as a medication for deep sedation. Propofol has been widely used due to its rapid onset of action within 2-3 min, short half-life, good amnesic potential, and increasing physician familiarity as a preferred drug for inducing sedation in a cardiovascular setting. Its side effects include bradycardia, hypotension, respiratory depression, hypertriglyceridemia, and propofol infusion syndrome. Still, no RCTs have been found [2,9,16].

#### 3.2.4. Other Treatments

The last of the categories are the others, including PCI and SGB. Coronary artery disease is prevalent in individuals with different causes of OHCA, especially in individuals presenting with shockable rhythms of VF/pVT. The current evidence suggests that early access to the CCL in patients resuscitated from VF/pVT cardiac arrest is associated with 2- to 3-fold higher functionally favourable survival rates than more conservative approaches with late or no access to the CCL [10]. Our patient was highly suspected of having CAD from the ECG and his history, so we sent him to the CCL as soon as possible. SGB has been used for the treatment of electrical storms. Theoretically, the majority of efferent sympathetic outflow to the heart travels through the stellate ganglion. Blocking the stellate ganglion may attenuate noradrenaline signalling and may suppress refractory VF/pVT, but no randomized clinical trials have identified SGB as suitable for refractory VF/pVT OHCA. SGB is not currently included in the AHA 2017 guidelines for the management of patients with ventricular arrhythmias [4].

## 4. Conclusions

We report a case of a STEMI patient suffering from refractory ventricular arrhythmia, which was treated successfully by a combination of multidisciplinary therapies. Refractory VF/pVT has a high mortality rate and is challenging to treat, and there is no single technique to manage it that has a promising effect. Emergency physicians may consider using multiple treatment methods as often as possible to treat refractory VF/pVT, to increase the likelihood of patient survival.

## Figures and Tables

**Table:** 

Category	Modality	Possible Mechanisms	Reference
Electrical management	1. Conventional treatment	Successful defibrillation affects most of the heart, resulting in too-little remaining heart muscle continuing the arrhythmia.	[7]
	2. DSD	Higher energy to overcome the increasing defibrillatory threshold. The first shock lowers the defibrillation threshold, thus increasing the second shock’s success. Alternate vectors of defibrillation provided by the second set of pads may be more likely to stop the myocytes fibrillating.	[4]
Mechanical support	1. CPR	Restores the partial flow of oxygenated blood to the brain and heart.	[7]
	2. ECMO	Normalizes perfusion reliably and provides cardiopulmonary support, to facilitate identification and treatment of the most common cause of refractory arrest (i.e., ACS).	[8]
Drugs	1. Amiodarone	Class III antiarrhythmic drug: blocks potassium rectifier currents that are responsible for the repolarization of the heart during phase 3 of the cardiac action potential.	[2]
	2. Lidocaine	Class Ib antiarrhythmic drug: blocks fast sodium channels responsible for the rapid phase-0 depolarization of the cardiac action potential in non-nodal tissue.	[2]
	3. Epinephrine	Stimulates alpha-adrenergic receptors, resulting in peripheral vasoconstriction, to increase coronary perfusion.	[1]
	4. Beta-blocker	Counteracts the negative beta-adrenergic effects of epinephrine to decrease the sensitivity of the myocardium to arrhythmias.	[3]
	5. Deep Sedation (Propofol)	Reduces sympathetic activity and enhance vagal tone. Direct effect on the cardiomyocyte through changes in protein kinase C translocation to different targets in the cell.	[9]
Miscellaneous	1. PCI	Restores myocardial blood flow.	[10]
	2. SGB	Blocks the stellate ganglion to attenuate noradrenaline signalling (efferent sympathetic outflow) to the heart.	[4]

Abbreviation: DSD—double sequential defibrillation; CPR—cardiopulmonary resuscitation; ECMO—extracorporeal membrane oxygenation; ACS—acute coronary syndrome; PCI—percutaneous coronary intervention; SGB—stellate ganglion block.

**Table 1:** Blood biochemistry data.

Parameters	Results	Normal Values
White blood cell count (/µL)	10,480	4800–10,800
Haemoglobin (g/dL)	13.1	14–18
Platelet count (/µL)	260,000	130,000–400,000
Mean corpuscular volume (fL)	90.7	80–94
BUN (mg/dL)	25.5	6–24
Creatinine (mg/dL)	1.63	0.5–1.4
Sodium (mEq/L)	138.9	137–145
Potassium (mEq/L)	3.9	3.1–5.3
GOT (mEq/L)	21.7	10–30
GPT (mg/dL)	18.2	2–32
CPK (U/L)	160	13–167
CRP (mg/dL)	1.04	<0.5
Hs-Troponin-I (pg/mL)	<10	<34.2

Abbreviations: BUN—blood urea nitrogen; GPT—glutamyl pyruvate transaminase; GOT—glutamyl oxaloacetic transaminase; CPK—creatine phosphokinase; CRP—C-reactive protein; Hs-Troponin-I—high-sensitivity troponin-I.

**Table 2:** The strategies of refractory ventricular arrhythmia management.

Category	Modality	Possible Mechanisms	Reference
Electrical management	1. Conventional treatment	Successful defibrillation affects most of the heart, resulting in too-little remaining heart muscle continuing the arrhythmia.	[7]
	2. DSD	1. Higher energy to overcome the increasing defibrillatory threshold. 2. The first shock lowers the defibrillation threshold, thus increasing the second shock’s success. 3. Alternate vectors of defibrillation provided by the second set of pads may be more likely to stop the myocytes fibrillating.	[4]
Mechanical support	1. CPR	Restores the partial flow of oxygenated blood to the brain and heart.	[7]
	2. ECMO	Normalizes perfusion reliably and provides cardiopulmonary support, to facilitate identification and treatment of the most common cause of refractory arrest (i.e., ACS).	[8]
Drugs	1. Amiodarone	Class III antiarrhythmic drug: blocks potassium rectifier currents that are responsible for the repolarization of the heart during phase 3 of the cardiac action potential.	[2]
	2. Lidocaine	Class Ib antiarrhythmic drug: blocks fast sodium channels responsible for the rapid phase-0 depolarization of the cardiac action potential in non-nodal tissue.	[2]
	3. Epinephrine	Stimulates alpha-adrenergic receptors, resulting in peripheral vasoconstriction, to increase coronary perfusion.	[1]
	4. Beta-blocker	Counteracts the negative beta-adrenergic effects of epinephrine to decrease the sensitivity of the myocardium to arrhythmias.	[3]
	5. Deep Sedation (Propofol)	1. Reduces sympathetic activity and enhance vagal tone. 2. Direct effect on the cardiomyocyte through changes in protein kinase C translocation to different targets in the cell.	[9]
Miscellaneous	1. PCI	Restores myocardial blood flow.	[10]
	2. SGB	Blocks the stellate ganglion to attenuate noradrenaline signalling (efferent sympathetic outflow) to the heart.	[4]

Abbreviation: DSD—double sequential defibrillation; CPR—cardiopulmonary resuscitation; ECMO—extracorporeal membrane oxygenation; ACS—acute coronary syndrome; PCI—percutaneous coronary intervention; SGB—stellate ganglion block.

## Data Availability

The data underlying this article can be shared upon reasonable request to the corresponding author.
